# Structural Modulation of Gut Microbiota during Alleviation of Suckling Piglets Diarrhoea with Herbal Formula

**DOI:** 10.1155/2017/8358151

**Published:** 2017-12-24

**Authors:** Cui Liu, Chao Zhang, Weijie Lv, Limin Chao, Zengquan Li, Dayou Shi, Shining Guo

**Affiliations:** ^1^College of Veterinary Medicine, South China Agricultural University, Guangzhou, China; ^2^Guangdong Technology Research Center for Traditional Chinese Veterinary Medicine and Nature Medicine, Guangzhou, China

## Abstract

To determine whether the traditional Chinese herbal formula of Shen Ling Baizhu (SLB) could modulate the composition of the gut microbiota and alleviate diarrhoea in suckling piglets, twenty-four newly born piglets (Large White × Landrace × Duroc) were selected and allocated to 4 groups (control group and experimental groups I, II, and III) randomly. Faecal microbiome composition was assessed by 16S rRNA gene 454-pyrosequencing. The result indicated that experimental groups I and II exhibited significantly different gut microbiota from the control group. Most notably, the genera* Lactobacillus* and* Bifidobacterium *were significantly elevated in experimental group II compared with the control group (*P* < 0.05).* Collinsella* and* Faecalibacterium *were also enhanced in experimental group II compared with the control group (*P* < 0.05). The results showed that SLB treatment could modulate the gut microbiota composition of suckling piglets, enriching the amount of beneficial bacteria in particular. The observed changes in the gut microbiota could provide the basis for further research on the pharmacological mechanism of the tested Chinese herbal formula.

## 1. Introduction

The growth and development of suckling piglets are critical processes in pigs. Sudden changes in psychology, environment, nutrition, and many other aspects are serious problems faced by piglets after weaning. Such changes can cause great stress to piglets, leading to disorders of physiological function and generating stress syndrome. Furthermore, these problems can decrease appetite and growth rates and increase the rate of diarrhoea [1, 2]. Diarrhoea occurring at high rates represents one of the most serious diseases in pigs [3] and can result in huge economic losses in the pig industry.

The gut microbiota may play a vital role in controlling diarrhoea [4, 5]. For example* Lactobacillus rhamnosus* GG or* Saccharomyces boulardii* can prevent antibiotic-associated diarrhoea and* S*.* boulardii* can prevent* Clostridium difficile*-associated diarrhoea [6]. A recent study indicated that the gut microbiota associated with Porcine epidemic diarrhoea (PED) significantly provided an insight into the pathology and physiology of PED [4]. Another study showed the* Clostridium difficile* infection (CDI) to be the most common cause of nosocomial diarrhoea and indicated that probiotics may be effective for secondary prevention in patients with recurrent CDI [7]. A more recent comparative analysis of the faecal microbiota of irritable bowel syndrome (IBS) patients and healthy subjects showed that the faecal microbiota (such as* Coprococcus*,* Collinsella,* and* Coprobacillus*) was significantly altered in IBS patient [8].* Collinsella* is a genus of short-chain fatty acid producers. The severity of postweaning diarrhoea is associated with rotavirus; however* Escherichia coli *can be reduced through dietary treatment using* Bifidobacterium lactis *HN019, possibly via a mechanism that enhances immune-mediated protection [9], while the probiotic* Escherichia coli* strain Nissle 1917 can prevent the effect of toxigenic EcA in the pig small intestine according to a previous study [10]. Taken together these studies revealed that gut microbiota may causatively contribute to diarrhoea.

To prevent diarrhoea in weaned piglets, adding antibiotics to the basal diet is a common method in pig production systems. However, the abuse of antibiotics not only results in drug residues in animals and bacterial drug resistance but also represents a serious threat to human health. A Chinese herbal formula (CHF) is a pure daily medicine consisting of single or multiflavoured herbs produced from natural medicinal plants, and traditional Chinese veterinary theory is the underlying theoretical basis [11]. Recent research has indicated that many ingredients in these herbs are only aimed at supporting the host's gut microbiota [12]. Shen Ling Baizhu (SLB), which is recorded in the “Taiping benevolent dispensary,” is a classic prescription for invigorating the spleen and strengthening qi and can reportedly be used in the treatment of functional dyspepsia and irritable bowel syndrome [13, 14]. It has been shown that SLB has a bidirectional regulatory effect on improving gastrointestinal motility [15] and can foster anaerobic bacteria and inhibit aerobic bacteria [16]. However, the modulation of gut microbiome of SLB treatment on suckling piglets diarrhoea has not been explored or discussed in the literature.

In this study, twenty-four newly born full-parity piglets of similar weight were selected to evaluate the efficacy and safety of SLB in the prevention and treatment of diarrhoea. After 5 weeks of treatment, we examined the structural alterations of the gut microbiome in response to the SLB treatment for the alleviation of diarrhoea.

## 2. Materials and Methods

### 2.1. Preparation of SLB

The TCM formula of SLB powder comprises ten herbs which were purchased from qualified suppliers based on standards specified in the Chinese Pharmacopoeia (Guangzhou, China). The original formula contains* Nelumbo *(500 g),* Coix* (500 g),* Amomum *(500 g),* Platycodon *(500 g),* Dolichos *(750 g),* Poria *(1000 g),* Ginseng *(1000 g),* Glycyrrhiza* (1000 g),* Atractylodes* (1000 g), and* Dioscorea* (1000 g). The herb materials were mixed and powdered by grinder. The ingredients and actions of the formulation are listed in Table 1.

### 2.2. Animal Ethics Statement

The pigs were provided by Minxin Animal Husbandry Co., Ltd., in Guangzhou, Guangdong. All experimental procedures in this study were approved by the Animal Ethics Committee of the South China Agricultural University (Guangzhou, China). The care and use of the animals were performed according to the Guidelines for Animal Experiments of South China Agriculture University (Guangzhou, China), and all efforts were made to minimize the number of animals used and their suffering.

### 2.3. Study Design

Twenty-four newly born full-parity piglets of similar weight were selected for this experiment, and they were randomly divided into 4 groups (*n* = 6, male and female): a control group, experimental group I, experimental group II, and experimental group III. The piglets in the control group were fed with conventional feed throughout the experimental period. In experimental group I, the diets of the sows were supplemented with 0.3% SLB from the postpartum period to the end of lactation. In experimental group II, the piglets had free access to Creep Feed with 0.3% SLB supplementation from birth to weaning. In experimental group III, the piglets had free access to Creep Feed with 0.3% SLB 3 days before weaning (the ingredient and nutrition composition of basal diet of suckling piglets and lactating sows were revealed as Tables S1 and S2). The feces of the piglets were observed, the number of piglets with diarrhoea was counted, and the rate of diarrhoea was calculated every day for the period from 7:30 to 19:00.

Faecal samples were collected from 24 piglets at 3, 6, 9, 14, 28, 32, and 35 days of age. All samples were preserved in an ice box, transported back to the university, and then stored at −40°C.

### 2.4. 16S rRNA Gene Sequence Analysis in Faecal Samples

Total metagenomic DNA was extracted from individual faecal samples using a TIAamp DNA Stool Mini Kit (Tiagen, DP328-02) according to the manufacturer's recommendations. To increase the DNA yield, an additional bead beating step was included, and the initial lysis temperature was increased from 70 to 95°C [17]. DNA quantification and quality were assessed by electrophoresis on a 1% (wt/vol) agarose gel and using a NanoDrop 3300 spectrophotometer [18].

DNA concentrations were diluted to a final concentration of 20 ng/*μ*L for PCR amplification of the V4 hypervariable regions of the 16S rRNA gene. The primers selected to amplify the V4 region included the forward primer 5′-AYTGGGYDTAAAGNG-3′ and the reverse primer 5-TACNVGGGTATCTAATCC-3. Each primer also included “barcode” sequences to facilitate the sequencing of products in the Roche 454 GS FLX+ system (454 Life Sciences, USA). The fusion primer sequences were 5′-454adapter-mid- CCTACGGGAGGCAGCAG-3′ (forward) and 5′-454adapter-mid- CCTACGGGAGGCAGCAG-3′ (reverse). The PCR reaction system is shown in Table S3. Each set of PCR reactions contained a negative control, in which the template DNA was replaced with sterile double-distilled water, and a positive control containing previously amplified faecal microbial DNA. The PCR conditions were as follows: an initial denaturation step at 98°C for 3 min, followed by 25 cycles at 98°C for 30 s, 50°C for 30 s, and 72°C for 30 s. A final 5 min extension step was performed at 72°C. The PCR amplification products were separated by electrophoresis through a 1.5% (wt/vol) agarose gel, extracted from the gel, and purified using the Axy-Prep DNA Gel Extraction Kit (Axygen, AP-GX-500). The quality of the products was assessed using a NanoDrop 3300 spectrophotometer [18]. Only PCR products without primer dimers and contaminant bands were used for pyrosequencing. Emulsion barcoding of the V4 amplicons was performed according to the “em-PCR Amplification Method Manual-Lib L,” and sequencing was performed using a 454 GS FLX+ (454 Life Sciences, USA) according to the “Sequencing Method Manual” [19].

### 2.5. Bioinformatics and Statistical Analyses

High-quality sequences were obtained using QIIME [20] and MOTHUR [21, 22]. Sequence clustering and OTU delineation by QIIME were performed as described previously [22, 23]. Representative sequences of operational taxonomic units (OTUs) and their relative abundance were employed to calculate the rarefaction analysis diversity index using QIIME [20]. In addition, community richness indexes, including the Chao index and the Shannon index, were obtained using MOTHUR (Table S4). The resultant phylogenetic tree and the table of the relative abundance of representative sequences of OTUs were used for Beta diversity analysis, including a principal coordinates analysis (PCoA), and the species community structure diagram was generated using MetaPhlAn [24]. Principal components analysis (PCA) was performed with R (3.0.2). According to the statistical data of the relative abundance at the two levels of the Taxonomy-based analysis at the phylum and genus level, data were expressed as means ± standard deviation (SD). Multigroup comparisons (the LSD test, Bonferroni method to correct the *P* value) were carried out by analysis of variance (ANOVA) with SPSS 20.0. Values of *P* < 0.05 were considered statistically significant. Functional genes prediction analysis (PICRUSt) based on the OTU with their relative abundance associated with human diseases, cellular processes, environmental information processing, genetic information processing, human diseases, metabolism and organismal systems, and the statistical analysis method was the same as above.

## 3. Results

### 3.1. SLB Significantly Reduced Diarrhoea Rate of Piglets

**Figure 1:**
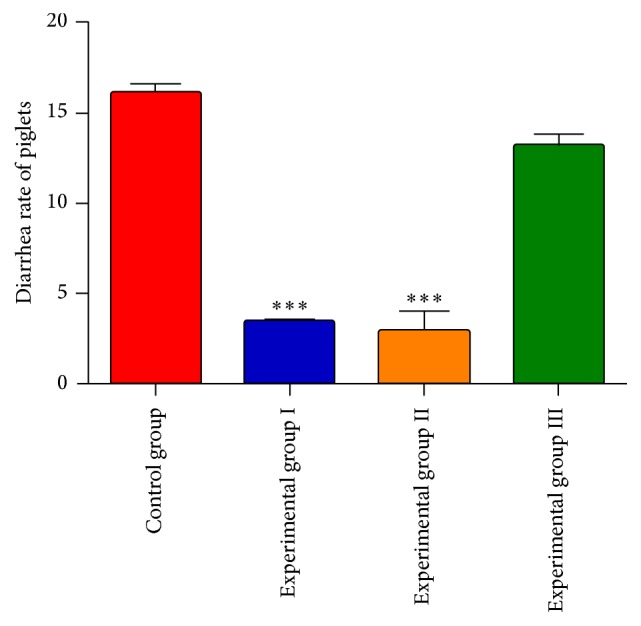
SLBS significantly reduced the diarrhoea rate in piglets. Experimental group I (*n* = 6), experimental group II (*n* = 6), experimental group III (*n* = 6), and the control group (*n* = 6). Data are presented as the mean ± SD. ^*∗∗∗*^*P* < 0.01 versus experimental group III and the control group using SPSS 20.0.

In the vivo test, the diarrhoea rate of 24 piglets was analysed as shown in Figure 1. After 5 weeks of treatment, SLB significantly reduced the diarrhoea rate in piglets. Experimental groups I and II showed a significant reduction of the diarrhoea rate compared with experimental group III and the control group (*P* < 0.01). A reduction of the diarrhoea rate was also observed in experimental group III; however, this reduction was not significant (Figure 1).

### 3.2. Overall Structural Modulation of Gut Microbiota after SLB Treatment

First, we used the high-throughput technology of bar-coded pyrosequencing to monitor the structural changes in the gut microbiota in the four groups before and after SLBS treatment. 14,403,422 high-quality sequences were obtained, with an average of 85,734 per sample. An identity of 97% was used as the cut-off, and 82,971 OTUs were obtained from 168 samples (Figure S1, Table S4). The Shannon curve indices all reached stable values, revealing that most of the bacterial diversity in these communities was covered.

**Figure 2:**
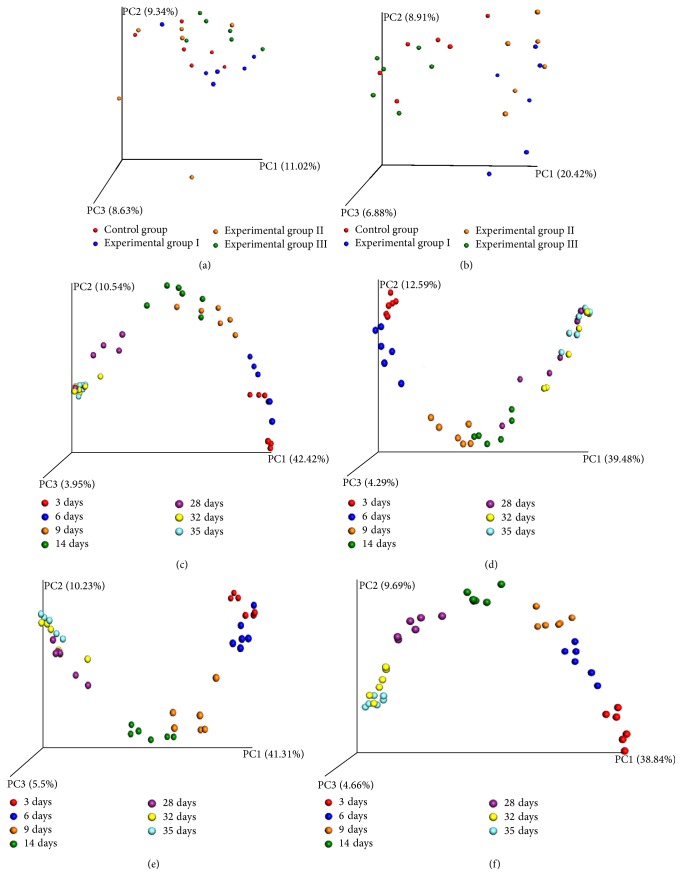
Principal coordinates analysis (PCoA) of the gut microbiota. (a) PCoA of the gut microbiota of 9-day-old piglets in different groups. (b) PCoA of the gut microbiota of 28-day-old piglets in different groups. (c) PCoA of the gut microbiota of piglets of various ages in experimental group I. (d) PCoA of the gut microbiota of piglets of various ages in experimental group II. (e) PCoA of the gut microbiota of piglets of various ages in experimental group III. (f) PCoA of the gut microbiota of piglets of various ages in the control group.

Unweighted UniFrac PCoA was employed to discriminate the microbiota composition of the different groups based on evolutionary distance. All of the samples from 9-day-old piglets were combined (Figure 2(a)). However, all of samples fell into three clusters. In addition, the samples from the control group and experimental group III exhibited a tendency to move closer together, as did the samples from experimental group I and experimental group II (Figure 2(b)). All of the samples showed significant changes with the change in the age of the piglets in all groups. Moreover, the samples from 32-day-old and 35-day-old piglets fell into one cluster in all groups (Figures 2(c), 2(d), 2(e), and 2(f)).

**Figure 3:**
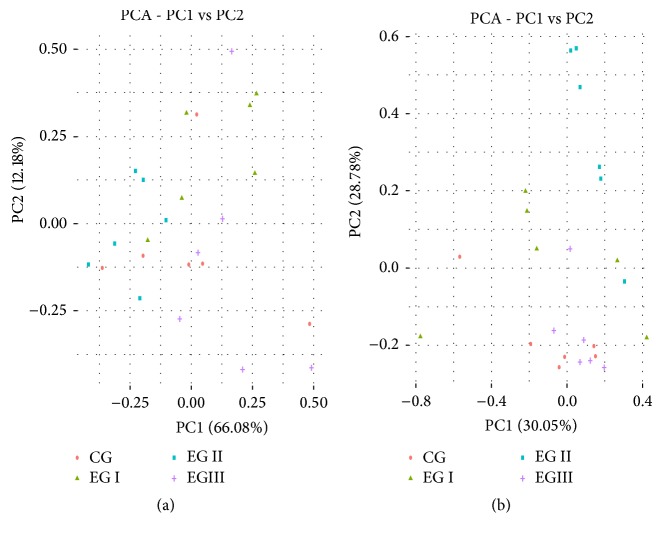
Principal components analysis (PCA) of the gut microbiota. (a) PCA of the gut microbiota of 9-day-old piglets in different groups. (b) PCA of the gut microbiota of 28-day-old piglets in different groups. CG: Control group. EGI: Experimental group I. EGII: Experimental group II. EGIII: Experimental group III.

PCA was used to reveal the relationship between large samples and multivariate data. All samples from 9-day-old piglets displayed a mixed distribution and did not fall into a single cluster (Figure 3(a)). Although the samples from 28-day-old piglets in experimental group I and experimental group II were dispersed, they were completely separated from the control group. In contrast, the samples in experimental group III were situated close together (Figure 3(a)).

### 3.3. Key Phylotypes of Gut Microbiota Modulated by SLB

**Figure 4:**
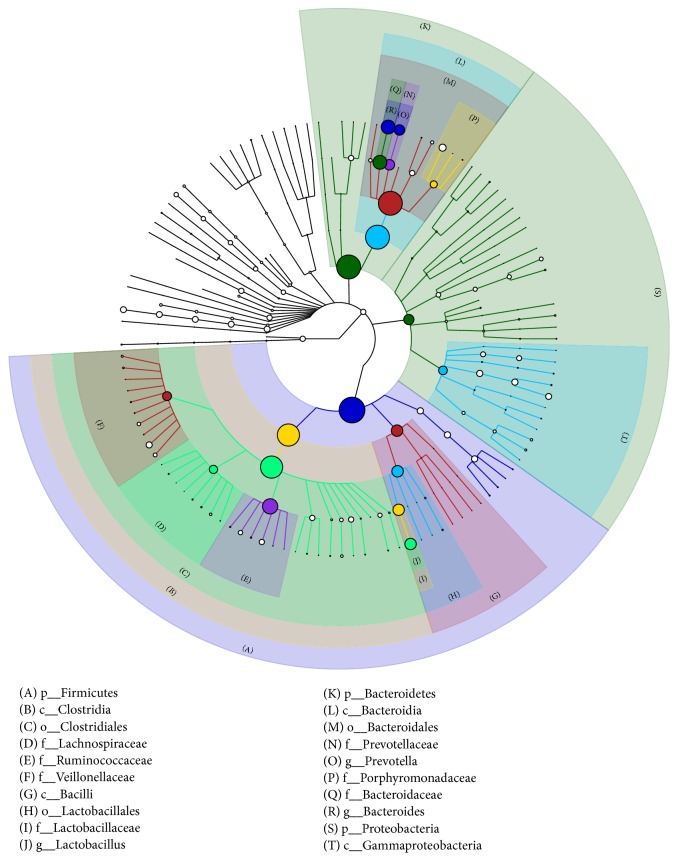
Community structure diagram of the gut microbiota of piglets. The size of the nodes reflects the species abundance at the corresponding species level, and the level of the first 10 of the species is identified in the map.

**Figure 5:**
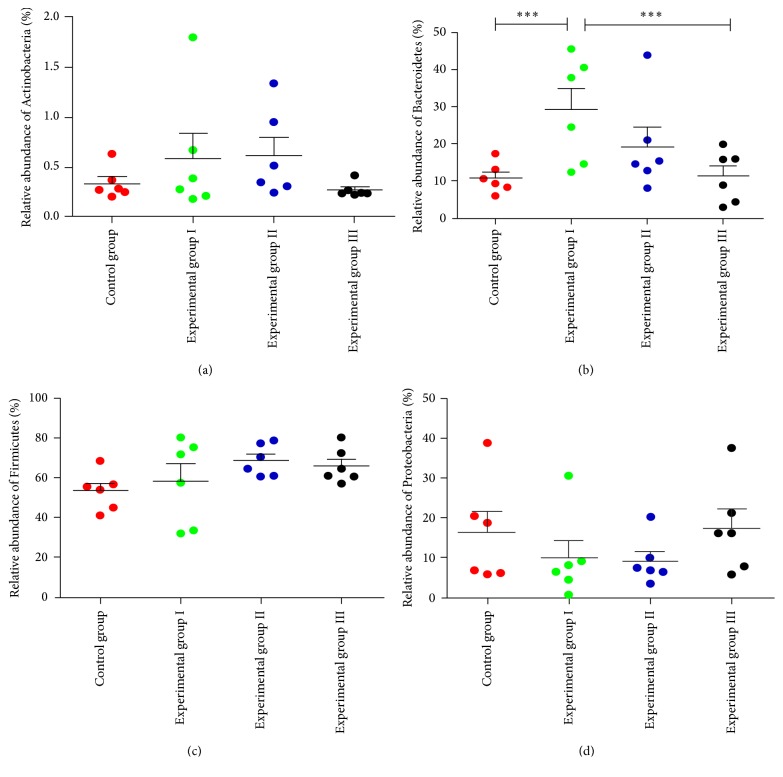
Taxonomy-based analysis at the phylum level in 32-day-old piglets. In each of the panels, the analysis result at the phylum level was indicated by the label on the *y*-axis. ^*∗∗∗*^*P* < 0.01 means the difference was extremely significant by the Multigroup comparisons (the LSD test) which were carried out by analysis of variance (ANOVA) with SPSS 20.0.

Based on the species community structure diagram, it can be observed that Firmicutes, Bacteroidetes and Proteobacteria represented the most important components of the intestinal microbiota of the piglets (Figure 4). Taxonomy-based analysis at the phylum level showed that Actinobacteria (Figure 5(a)) and Firmicutes (Figure 5(c)) were increased in experimental group I and experimental group II, while Proteobacteria (Figure 5(d)) were decreased in experimental group I and experimental group II, although this decrease was not significant. Nevertheless, there were significantly fewer Bacteroidetes in the control group and experimental group III versus experimental group I (Figure 5(b)).

**Figure 6:**
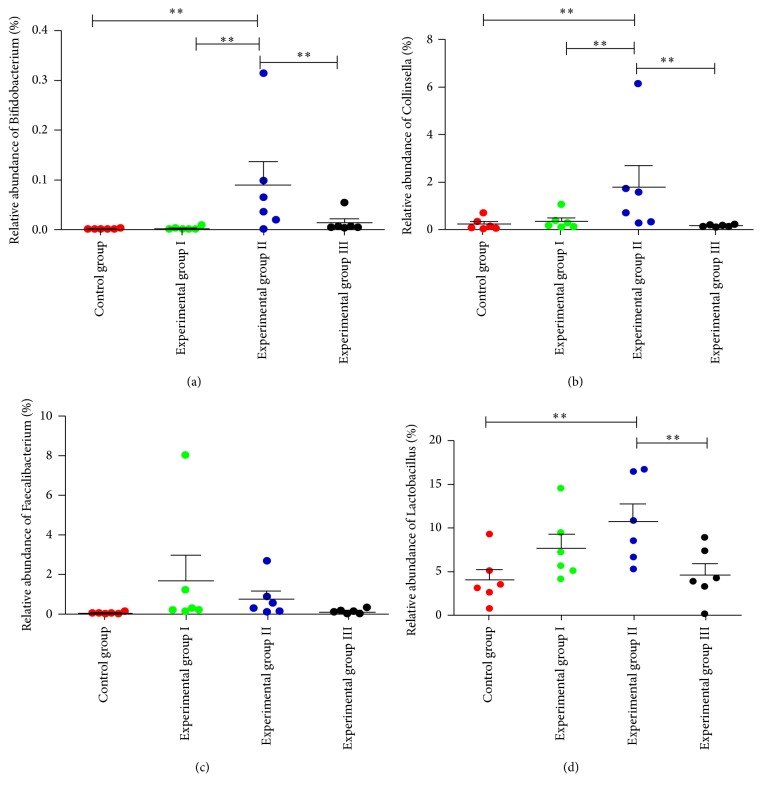
Taxonomy-based analysis at the genus level in 32-day-old piglets. In each of the panels, the analysis result at the genus level was indicated by the label on the *y*-axis. ^*∗∗*^*P* < 0.05 means the difference was significant by the Multigroup comparisons (the LSD test) which were carried out by analysis of variance (ANOVA) with SPSS 20.0.

Taxonomy-based comparison at the genus level further showed that, in each group, the genera* Prevotella* and* Bacteroides* from phylum Bacteroidetes and the genera* Eubacterium, Blautia, Coprococcus, Oscillospira, Lactobacillus, *and* Faecalibacterium* from phylum Firmicutes and the genera* Bifidobacterium and Collinsella *from phylum Actinobacteria were predominant (each genus exhibited a relative abundance > 0.1%). The genera* Prevotella, Bacteroides, *and* Eubacterium* were decreased in all experimental groups, while* Blautia, Coprococcus, Oscillospira, Faecalibacterium, Lactobacillus, Bifidobacterium, *and* Collinsella* were increased (Table S5, Figure 6). Additionally, the beneficial gut microbiota* Bifidobacterium* and* Lactobacillus* were significantly increased in experimental group II (*P* < 0.05) (Figures 6(a) and 6(d)).* Collinsella *was also significantly increased in experimental group II (*P* < 0.05) (Figure 6(b)).

### 3.4. Functional Gene Analysis

**Figure 7:**
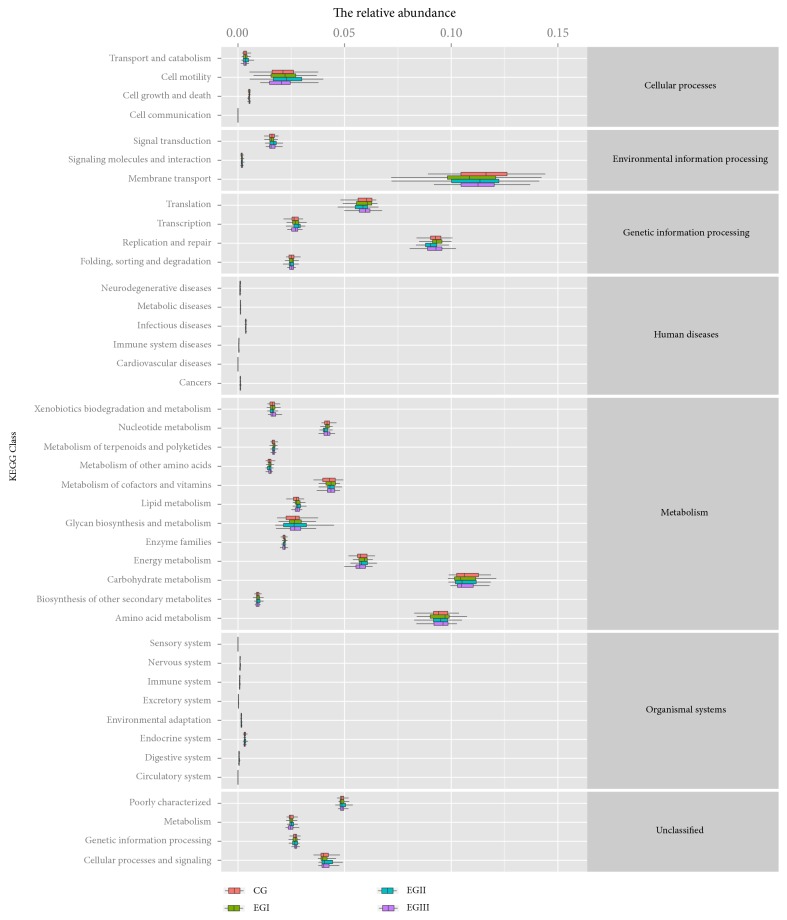
KEGG statistics for the functional genes of 32-day-old piglets. The horizontal coordinates represent the relative abundance of functional genes in different groups; the vertical coordinates represent the functions of genes. CG: Control group. EGI: Experimental groupI. EGII: Experimental group II. EGIII: Experimental group III.

Based on the analysis of metabolism genes (Figure 7), compared with the control group, the relative abundance of functional genes in the following categories was increased in experimental group I and experimental group III: nucleotide metabolism, the metabolism of terpenoids and polyketides, the metabolism of other amino acids, the metabolism of cofactors and vitamins, lipid metabolism, glycan biosynthesis and metabolism, enzyme families, energy metabolism, carbohydrate metabolism, biosynthesis of other secondary metabolites, and amino acid metabolism. Additionally, the metabolism of cofactors and vitamins, lipid metabolism, amino acid metabolism, and the metabolism of the above functional genes were also enhanced in experimental group II. The difference in three categories of functional genes was extremely significant among the following groups (Figure S2) (*P* < 0.01). In the immune system disease of functional genes, the control group was significantly higher than experimental group III (*P* < 0.01). In the energy metabolism of functional genes, experimental group I is higher than experimental group III (*P* < 0.01). In the Cell Growth and Death of functional genes, experimental group II was higher than the control group and experimental group I (*P* < 0.01) (Figure S2).

## 4. Discussion

SLB conferred meaningful prevention of diarrhoea in piglets compared with the control group. We observed a slightly decreased rate of diarrhoea in experimental groups I and II. This finding was consistent with a previous study in which SLB was added to the diets of pregnant sows 20 days before delivery, and the diarrhoea rate in piglets was effectively controlled [25]. Other studies have suggested that SLB is a safe and effective treatment for enteral nutrition-related diarrhoea [26] and that it can slightly alleviate diarrhoea associated with irritable bowel syndrome [27]. Moreover, SLB can effectively decrease the amount of stool produced and improve faecal characteristics in chronic diarrhoea patients [28]. These data indicate that this traditional Chinese herbal formula is effective for diarrhoea control and that it may be a promising candidate for use in diarrhoea management.

We observed an altered microbiota composition induced by SLB. In other studies, SLB has been shown to restore damaged intestinal tissue by enhancing beneficial intestinal bacterial species and then restoring the bacteria to a state of equilibrium [29]. Previous studies have also shown that SLB is capable of modulating the gut microbiota by supporting the healthful genus* Bifidobacterium* and inhibiting major drug-resistant strains [16]. However, these studies have not provided complete profiling of the gut microbiota about SLB treatment on suckling piglets diarrhoea. In the present study, using the high-throughput technology of bar-coded pyrosequencing, along with unweighted UniFrac PCoA analysis and PCA data, we observed significant structural changes in the gut microbiota in experimental group I and experimental group II compared with the control group. The result indicated that experimental groups I and II exhibited significantly different gut microbiota from the control group. Most notably, the genera* Lactobacillus* and* Bifidobacterium *were significantly elevated in experimental group II compared with the control group (*P* < 0.05).* Collinsella* and* Faecalibacterium *were also enhanced in experimental group II compared with the control group.

The intestinal probiotic genera* Bifidobacterium* and* Lactobacillus *were significantly enriched by SLB in our study. Probiotics may be effective for secondary prevention in patients with recurrent* Clostridium difficile*-associated diarrhoea through maintenance of the normal gastrointestinal flora [7]. In addition, the dietary supplements,* Bifidobacterium lactis *420, have beneficial impacts on intestinal barrier integrity [30]. Moreover, the diarrhoea of weaned piglets challenged by rotavirus was shown to be alleviated via the inhibition of virus multiplication, and jejunal mucosal barrier function was improved through supplementing* Lactobacillus rhamnosus GG* in diets, possibly due to decreasing the apoptosis of jejunal mucosal cells [31]. Additionally, the probiotic species* Lactobacillus casei *Zhang can reduce LPS/GalN-induced proinflammatory cytokine levels [32], and* Lactobacillus reuteri* can decrease pathogen colonization and subsequently improve paediatric gut health when administered early [33]. The short-chain fatty acid (SCFA) producers* Faecalibacterium *and* Collinsella* were also enhanced by SLB in our study, and the increase in* Collinsella *reached a significant level. In other studies, SCFAs have shown promise for treating inflammatory bowel disease [34], mediating intestinal barrier function [35], and increasing anti-inflammatory cytokine secretion [36]. SCFAs are also related to water inhibition and electrolyte absorption, and they are a preferred energy source for the colonic epithelium [37]. However, high* Collinsella* numbers have been detected in healthy humans compared with patients with irritable bowel syndrome (IBS) [8], Crohn's disease [38], diarrhoea [39], and nonalcoholic fatty liver disease [40]. Additionally, as a potential probiotic,* Faecalibacterium prausnitzii *can improve the gastrointestinal health and growth of preweaned calves [41] and plays a pivotal role in treating the pathogenesis of colitis, inflammatory bowel disease, and Crohn's disease [42]. In the study,* Collinsella, Bifidobacterium,* and* Lactobacillus* were significantly enhanced in experimental group II, indicating that treatment of the sows with 0.3% SLB had a better effect on improving the intestinal microflora in suckling piglets through vertical transfer via breast milk [43]. Thus, the significant increase in beneficial bacterial due to treatment with SLB observed in our study may indicate some other unknown protective measures that warrant further investigation.

In conclusion, our study indicated that the structural gut microbiota of suckling piglets was modulated by the Chinese herbal formula SLB via both direct effects in suckling piglets and indirect effects in the suckling piglets through its effects on sows. In particular, this treatment increased the number of beneficial bacteria, such as* Bifidobacterium *spp.,* Lactobacillus *spp.,* Faecalibacterium *spp., and* Collinsella *spp., which may act directly or indirectly on the course of diarrhoea in the gut. However, the causal relationship between the microbiome and this disease is still unclear, requiring further investigation. Our study provides a theoretical basis for further investigations concerning the prevention of diarrhoea with SLB.

## Figures and Tables

**Table 1:** The ten Chinese herbs utilized in this study.

Latin names	Chinese names	Actions
*Nelumbo*	Lian Zi	Invigorate *Qi*, strengthen the Spleen, stop diarrhea
*Coix*	Yi Yi Ren	Strengthen the Spleen, promote diuresis, excrete Dampness and calm the mind
*Amomum*	Sha Ren	Promote Stomach *Qi *flow to improve appetite, facilitate digestion and stop vomiting
*Platycodon*	Jie Geng	Open the Lung to relieve cough, dissolve Phlegm, guide other herbs to the upper part of the body
*Dolichos*	Bai Bian Dou	Strengthen the Spleen, eliminate Dampness
*Poria*	Fu Ling	Strengthen the Spleen, induce diuresis, excrete Dampness
*Ginseng*	Ren Shen	Replenish the Source *(Yuan Qi)*, tonify the Spleen and Lung
*Glycyrrhiza*	Gan Cao	Harmonize the effects of other herbs
*Atractylodes*	Bai Zhu	Strengthen the Spleen, dry up Dampness, tonify *Qi *and promote diuresis
*Dioscorea*	Shan Yao	Tonify the Spleen and Lung

## Supplementary Materials

Supplementary Materials Table S1. The composition of basal diet of suckling piglets. Table S2. The composition of basal diet of lactating sows. Table S3. The PCR reaction system. Table S4. Richness and diversity estimation based on pyrosequencing analysis. Table S5. Structure modulate of the gut microbiota in genus level. Figure S1. Shannon curves for the gut microbiota of suckling piglets. Figure S2. The significance of KEGG statistics for the functional genes of 32-day-old piglets. In each of the panels, the analysis result of KEGG was indicated by the label on the *y*-axis. ^*∗∗*^*P* < 0.01 means the difference was extremely significant by the Multigroup comparisons (the LSD test) which were carried out by analysis of variance (ANOVA) with SPSS 20.0.
