# Supplementary material for: Structural Modulation of Gut Microbiota during Alleviation of Suckling Piglets Diarrhoea with Herbal Formula
Source: Evid Based Complement Alternat Med. 2017 Dec 24;2017:8358151. doi: 10.1155/2017/8358151 (PMC5757150; doi:10.1155/2017/8358151)
Supplement: Supplementary Materials — Table S1. The composition of basal diet of suckling piglets. Table S2. The composition of basal diet of lactating sows. Table S3. The PCR reaction system. Table S4. Richness and diversity estimation based on pyrosequencing analysis. Table S5. Structure modulate of the gut microbiota in genus level. Figure S1. Shannon curves for the gut microbiota of suckling piglets. Figure S2. The significance of KEGG statistics for the functional genes of 32-day-old piglets. In each of the panels, the analysis result of KEGG was indicated by the label on the y-axis. ∗∗P < 0.01 means the difference was extremely significant by the Multigroup comparisons (the LSD test) which were carried out by analysis of variance (ANOVA) with SPSS 20.0. [file 8358151.f1.pdf]

Supplementary materials

Table S1. The composition of basal diet of Suckling piglets

	Dry matter feed
Ingredients	
Expanded corn	30.00%
Corn	26.30%
Full-fat soybean	12.50%
Whey powder	12.00%
Fermented soybean meal	5.00%
Plasma protein powder	4.00%
Fish meal	5.00%
Soybean oil	2.00%
Monocalcium phosphhate	0.40%
Calcium carbonate	0.60%
Acidifiers	0.40%
Sodium butyrate	0.25%
Zinc oxide	0.35%
Premix	1.00%
Nutrients	
Digestible energy	3500Kcal·kg^{-1}
Crude protein	18.00%
Calcium	0.65%
phosphorus	0.45%
Lys	1.40%
Met	0.45%
Thr	0.91%
Try	0.24%

Table S2. The composition of basal diet of lactating sows

	Dry matter feed
Ingredients	
Corn	60.4%
Wheat bran	10.4%
Cottonseed meal	6.8%
Fish meal	5.1%
Soybean Meal	1.8%
bone meal	1.2%
salt	0.5%
Peanut cake	12.6%
Sweet Potato Vine	1.2%
Nutrients	
Digestible energy	2.61Kcal·kg^{-1}
Crude protein	13.8%
Lys	0.68%
phosphorus	0.67%

Table S3. The PCR reaction system

	Amount
Sterilized ultra pure water	8.75µl
Q5 Reaction Buffer（5×）	5.0µl
Q5 GC high Enhancer （5×）	5.0µl
dNTP（2.5mM）	2.00µl
Template（20ng/µl）	2.00µl
Primer F（10µM）	1.00µl
Primer R（10µM）	1.00µl
Q5 Polymerase(5U/ul)	0.25µl
Total amount	25.00µl

Table S4. Richness and diversity estimation based on pyrosequencing analysis

Groups		High-qualitysequences	OTUs	Chao1	Shannon
	CG	692555	1625	2079.99	21.60^{bB}
3 days	EGI	727690	1646	2064.45	24.12^{acA}
	EG II	636069	1602	1971.14	15.61^{c}
	EG III	557795	1551	2105.15	23.53^{acA}
	CG	343687	1859	2411.94^{a}	27.65^{aB}
6 days	EGI	409748	1546	1992.30^{b}	21.33^{b}
	EG II	529999	1590	2085.02^{ab}	20.23^{bA}
	EG III	483993	1646	1980.76^{b}	22.16^{b}
	CG	570239	2166	2732.54^{bc}	24.42
9 days	EGI	619916	2421	3114.85^{ac}	26.27
	EG II	584274	2609	2807.35^{a}	22.96
	EG III	586370	2365	3022.67^{ac}	23.40
	CG	553015	2573	4164.07^{aB}	26.40
14 days	EGI	584749	2609	3302.43^{aA}	24.58
	EG II	538471	3130	4115.29^{aB}	25.17
	EG III	568852	2819	3770.35^{a}	28.50
	CG	636746	3865	4758.73^{a}	33.05^{b}
28 days	EGI	711061	4414	5097.15^{ab}	35.69^{ab}
	EG II	579160	4278	5126.55^{ab}	35.42^{ab}
	EG III	782082	4531	5249.87^{b}	38.80^{a}
	CG	252219	3711	4483.68^{bc}	36.39^{ab}
32 days	EGI	758698	4818	5524.10^{aB}	39.77^{a}
	EG II	327423	4011	4837.60^{b}	37.48^{ab}
	EG III	239480	3539	4274.08^{bA}	33.47^{b}
	CG	277118	4046	4751.88	39.54^{ab}
35 days	EGI	270805	3660	4522.73	36.76^{b}
	EG II	278024	3844	4625.55	37.34^{ab}
	EG III	283502	4056	4795.13	39.94^{a}

Table S5. Structure modulate of the gut microbiota in genus level

Bacteria	Control group	Experimental groupI	Experimental group II	Experimental group III
Eubacterium	0.33±0.04	0.11±0.02	0.42±0.03	0.23±0.03
Prevotella	1.76±0.07	1.37±0.03	0.99±0.04	0.74±0.05
Bacteroides	2.40±0.08	2.48±0.07	4.23±0.17	1.13±0.05
Blautia	0.06±0.01	0.15±0.02	0.13±0.02	0.04±0.00
Coprococcus	0.37±0.07	0.66±0.04	0.37±0.02	0.53±0.06
Oscillospira	2.15±0.08	2.56±0.06	4.72±0.46	2.53±0.09

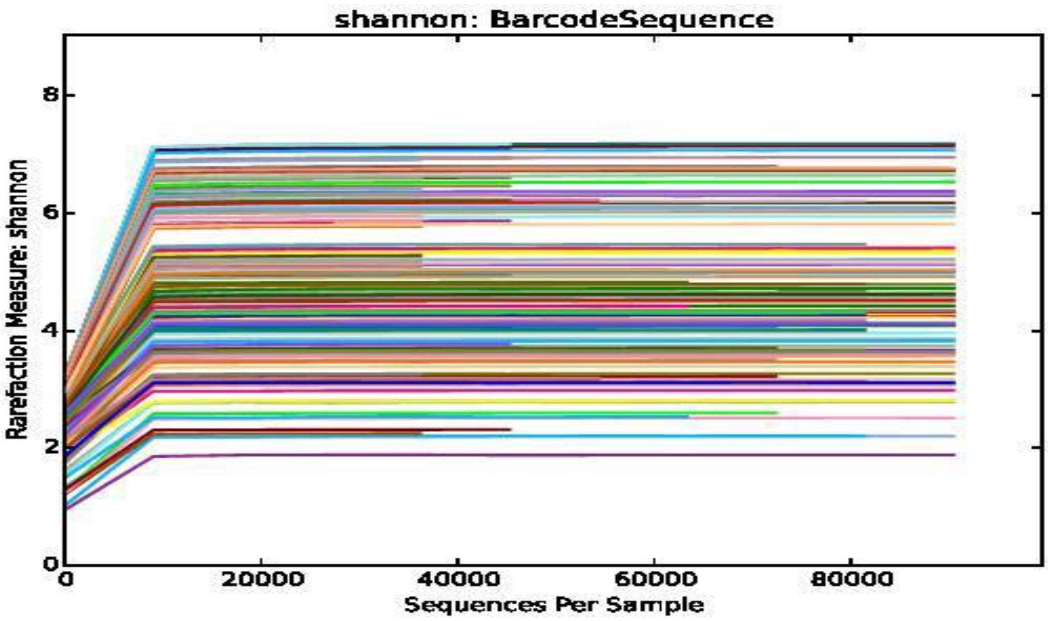

Fig. S1. Shannon curves for the gut microbiota of suckling piglets.

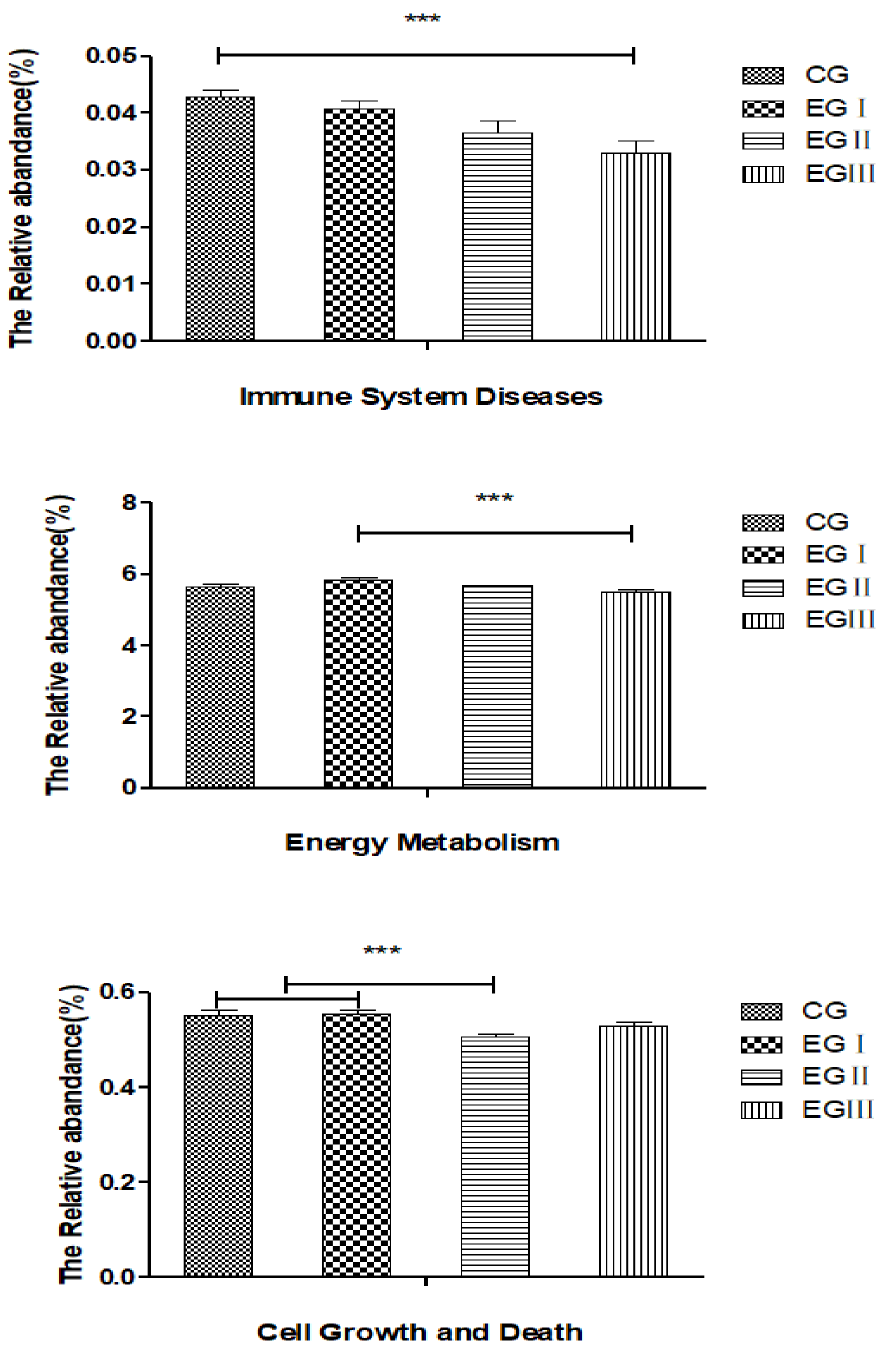

Fig. S2. The significance of KEGG statistics for the functional genes of 32-day-old piglets. In each of the panels, the analysis result of KEGG was indicated by the label on the y-axis. $^{**}P < 0.01$ means the difference was extremely significant by the Multigroup comparisons (the LSD test）were carried out by analysis of variance (ANOVA) with SPSS 20.0.
